# Effects of One-Hour Training Course and Spirometry on the Ability of Physicians to Diagnose and Treat Chronic Obstructive Pulmonary Disease

**DOI:** 10.1371/journal.pone.0117348

**Published:** 2015-02-23

**Authors:** Shan Cai, Li Qin, Lynn Tanoue, Anmei Hu, Xiujie Jia, Hong Luo, Yan Chen, Ping Chen, Hong Peng

**Affiliations:** 1 Department of Respiratory Medicine, The 2^nd^ Xiangya Hospital, Central South University, Changsha, Hunan, 410011, P.R. China; 2 Department of Internal Medicine, Yale University, New Haven, CT, 06520–8234, United States of America; Georgia Regents University, UNITED STATES

## Abstract

**Backgrounds:**

In China, the prevalence of chronic obstructive pulmonary disease (COPD) in persons 40 years of age or older is estimated at 8.2%, but this is likely a substantial underestimate.

**Methods:**

Eight secondary hospitals which didn’t have spirometries were chosen randomly in Hunan province of central south China. Physician subjects at these hospitals underwent a one-hour training course on the Chinese COPD guidelines. Physicians answered questionnaires assessing their knowledge of the guidelines before and after the training session. The mean correct scores of questionnaires were compared before and after training. Four out of the eight hospitals were given access to spirometry. Eligible patient subjects underwent spirometry testing prior to the physician visit. After seeing the patient, physicians were asked to answer a questionnaire relating to the diagnosis and severity of COPD. Physicians were then given the results of the spirometry, and asked to answer the same questionnaire. Physicians’ responses before and after receiving the spirometry results were compared.

**Results:**

225 physicians participated in the training session. 207 questionnaires were completed. Mean scores (out of 100) before and after the training were 53.1 ± 21.7 and 93.3 ± 9.8, respectively. 18 physicians and 307 patient subjects participated in the spirometry intervention. Based on spirometric results, the prevalence of COPD was 38.8%. Physicians correctly identified the presence of COPD without spirometric data in 85 cases (76.6%); this increased to 117 cases (97.4%) once spirometric data were available. Without spirometric data, physicians incorrectly diagnosed COPD in 38 patients; this decreased to 6 patients once spirometric data were available. Spirometric data also improved the ability of physicians to correctly grade COPD severity.

**Conclusions:**

Simple educational training can substantially improve physicians’ knowledge relating to COPD. Spirometry combined with education improves the ability of physicians to diagnose COPD and to assess its severity.

## Background

Chronic obstructive pulmonary disease (COPD) is the fourth leading cause of death in the world. Further increases in its prevalence and associated mortality are predicted in the coming decades. Cigarette smoking is the most common risk factor for this disease. China is the world’s largest cigarette consumer and producer[1]. In 2002, about 350 million Chinese adults reported having smoked at some point and 300 million reported being current smokers[2]. COPD is a growing health problem in China. The prevalence of COPD in China in persons 40 years of age or older has been estimated at 8.2%[3].

The Chinese government faces a monumental task in trying to provide medical services adequate to meet the basic needs of an immense population spread over a vast area. Preventive care is limited in most of the population. For cultural and economic reasons, most Chinese people do not visit a physician unless they are acutely ill, which leads to difficulty in screening for asymptomatic disease. Other studies have reported that COPD is under-diagnosed in China. In one of the largest hospitals in Zhejiang province in China, the percentage of COPD misdiagnosis was as high as 47.4% in the respiratory department, and 91.9% in other departments[4]. In another study, nearly two thirds of individuals with spirometric evidence of COPD had never been diagnosed with COPD, with the rate of COPD underdiagnosis in rural areas being much higher than in urban areas in China[5].

In China, hospitals are divided into three levels by the government according to physical size of the medical campus, the number of inpatient beds, the type of medical equipment, etc. The majority of the Chinese population (approximately 55%) receives its inpatient medical care at secondary hospitals[6]. In these hospitals, the educational level of the majority of physicians ranges from three to five years of medical college after secondary school training. The majority of secondary hospital physicians do not have formal medical residency training. In contrast, almost all tertiary hospital physicians have graduated from medical colleges, and have pursued formal medical residency training. Primary care physicians in urban secondary hospitals in China have demonstrated a severe lack of knowledge relating to COPD[7–8]. With regard to facilities, most secondary hospitals do not have advanced diagnostic medical equipment, such as pulmonary function testing machines or simple spirometers[9]. In comparison, all tertiary hospitals have pulmonary function testing capabilities.

Spirometry is defined as an essential tool in the diagnosis and severity evaluation of COPD in current guidelines[10]. The Chinese COPD guideline[11] is very similar to the GOLD guideline[12], especially the case definition, severity grading and treatment by stage. However, it is underutilized. In a study of general practitioners in Italy, spirometry was used to confirm COPD diagnosis by only 69.8% of physicians[13]. Most Chinese primary and secondary hospitals do not have ready access to spirometric equipment. Further, the knowledge base of physicians working in these settings may be limited. In light of the cigarette smoking epidemic in China, there is an urgent need to raise awareness of COPD among primary care physicians by providing educational interventions, and to provide tools such as spirometry to aid in establishing the diagnosis in patients at risk and rendering appropriate treatment. Access to spirometry, and a compatible educational program in its utilization were identified as a critical steps to improve the diagnosis and treatment of COPD[14].

In a previous study[15], we found that only approximately one third of secondary hospitals have spirometers. Physicians working in tertiary hospitals had better knowledge of the Chinese COPD guideline than those in primary and secondary hospitals. Since spirometry is a key part of diagnosis and is the metric upon which severity of illness is based, the difference in the availability of spirometry may inevitably contribute to different COPD diagnostic capabilities. The aims of this study were to measure the effects of a one-hour training course on physicians΄ knowledge of COPD, and to assess the effects of spirometry on the ability of physicians to diagnose COPD in secondary hospitals in China.

## Materials and Methods

### Study design and population

This is a prospective observational study to assess the knowledge of internal medicine physicians practicing in secondary hospitals relating to the Chinese COPD guideline[11], before and after an educational intervention of a one-hour training course. In Hunan Province in central south China, there are 44 tertiary hospitals, 292 secondary hospitals and 193 primary hospitals. 8 secondary hospitals without spirometers were selected by random number table. 4 of those secondary hospitals were randomly selected to participate in the second intervention to evaluate the effect of spirometry on the diagnosis and treatment of COPD. Physician subjects included in the educational intervention were internal medicine physicians working in the 8 secondary hospitals. Physician subjects participating in the second intervention must have participated in the first educational intervention. In the second intervention, patients who were registered in the ambulatory internal medicine clinics were recruited by a research assistant. Inclusion criteria for patients included (1) ≥ 50 years of age, and (2) smoking history ≥ 20 pack-years. These criteria were intended to enrich the likelihood of COPD in the patient population. Exclusion criteria for patients included: (1) pulmonary symptomd as the chief complaint for the ambulatory visit, or chronic pulmonary complaints that patients wished to discuss with the physician during the ambulatory visit; (2) a contraindication to administration of salbutamol, including documented allergy to salbutamol, history of severe arrhythmia, uncontrolled hyperthyroidism, or uncontrolled hypertension, and women who are pregnant; and (3) subjects unable to independently give informed consent. Exclusion criteria were intended to eliminate patients for whom spirometry might yield erroneous results, or who would be unable to accept treatment because of medical contraindications.

The study was approved by the Yale University Institutional Review Board and the Ethics Committee of the Second Xiangya Hospital. Internal medicine physicians who were willing to attend the one-hour COPD training course were invited to participate in the study, and signed written informed consent. Patient participants received information as to why the study was being performed, and were required to give written informed consent to participate in the study.

### Diagnostic procedures and criteria

The first intervention was a one-hour training course based on the Chinese COPD guideline[11], which is very similar to the GOLD guideline[12], especially the case definition, severity grading and treatment by stage. Physicians who were willing to attend the training were invited to participate in the study, and signed the written informed consent. The training session was delivered by a pulmonary specialist who had completed PhD level training and had a teacher certification. The educational content was based on the Chinese Guideline for Diagnosis and Treatment of COPD[14]. A questionnaire surveying the contents of the guideline was administered to physicians and used to test their knowledge about the content of the guideline before and after the training session. The questionnaires were administered by a research assistant. Individual scores of the questionnaire before and after the educational intervention were measured.

In the second intervention, a portable spirometer was placed at the ambulatory clinics in four secondary hospitals for one month immediately after educational intervention. A research assistant was assigned to the clinics to recruit and screen eligible outpatients. Eligible outpatient subjects who consented to participate were asked about respiratory symptoms and related medical history and then underwent spirometry and bronchodilator testing to confirm a diagnosis of COPD. Spirometry was measured pre- and 10–15 minutes post inhaled salbutamol, 400 ug. The spirometry tests were performed according to the guideline[16]. A note was placed in the patient’s chart to indicate to the examining physician that he/she was participating in this study. After the physician visit, the research assistant administered a questionnaire to the physician to determine whether or not the physician felt that the patient had COPD, and if so, what the severity of disease was. The physician was then given the patient’s spirometry results, and a second questionnaire was administered. Physicians’ responses before and after they read spirometry results were compared. The diagnostic standard of the presence or absence of COPD was determined by the criteria of the Chinese COPD guideline, with the criterion for confirmation of a diagnosis of COPD being a post-bronchodilator FEV_1_/FVC ratio of <0.7. The severity of airway obstruction was graded based on the post-bronchodilator FEV_1_ as follows: mild grade: FEV_1_ >80%; moderate grade: FEV_1_ 50–79%; severe grade: FEV_1_ 30–49%; very severe grade FEV_1_ <30%., based on the Global Obstructive Lung Disease (GOLD) criteria[12] and Chinese Guideline for diagnosis and treatment of COPD (revised edition 2007)[11].

### Treatment of COPD

Physicians had determined treatment for COPD, if any, before being given the spirometry results. After the results were made available, physicians could choose to change treatment. An assessment was made as to whether the treatment prescribed was adherent to the GOLD[12] and Chinese COPD guidelines[11]. The percentage of adherent (correct) treatment before and after the physician received the spirometry results were was compared.

### Development and distribution of questionnaire

There were two questionnaires used in the educational and spirometric inventions, respectively. We cooperated with a pulmonary expert advisory group to devise the questionnaires. The questionnaire used in the educational intervention was based on the Chinese COPD guideline, and included a total of 10 multiple choice questions. The specific questions related to included hallmark symptoms of COPD, pathogenesis of COPD, guideline criteria of airflow limitation, guideline criteria for severity grading, therapy for COPD including pharmacotherapy and oxygen, the common causes of acute exacerbation COPD (AECOPD) and indications for non-invasive ventilation. There was a total of 100 score for the 10 questions, which were weighted equally. The questionnaires are administered anonymously before and immediately after the educational intervention. Information on physician gender, age, medical education, and medical rank was collected. Physicians’ education in China is divided into three groups: high, median and low educational levels. High educational level is defined as PhD and master’s degree of medicine, median educational level as Bachelor degree of medicine, and low educational level as having graduated from junior medical college. Medical rank is also classified by high, median and low levels according to physician clinical experience and duration. Discussion, internet access, and other aides were not permitted while physicians answered the questionnaires.

The questionnaire used in the spirometry intervention was designed to assess the ability of physicians to diagnose and treat COPD patients, with and without spirometry results. This questionnaire contained three questions: 1) whether or not the patient had COPD, 2) if the answer to 1) was yes, what the severity grade was, and 3) what the appropriate treatment was for the patient according to the Chinese COPD guideline. The questionnaire was administered after the completion of the patient visit, and then readministered after the physician had been given the spirometry results.

### Data analysis

All data were input and analyzed by statistical product and service solutions 15.0. Descriptive statistics were conducted and evaluated to examine differences pre- and post-intervention. For the educational intervention, the correct rate of each question pre- and post education was compared with McNemar testing; the mean scores of questionnaires pre- and post-education were compared by paired T-test. For the spirometry intervention, the responses of the questionnaires before and after the physician viewing of spirometry results were compared with McNemar testing. For both interventions, α = 0.05; with a significant difference between two groups when the p-value is less than 0.05. The accuracy of physician diagnosis pre- and post-spirometry was compared by kappa testing. A kappa value of<0.01 was considered to represent less than chance agreement; 0.01–0.20, slight agreement; 0.21–0.40, fair agreement; 0.41–0.60, moderate agreement; 0.61–0.80, substantial agreement; and 0.81–1.0, almost complete agreement. Wilcoxon signed rank sum test was used to assess the impact of spirometry on physicians’ ability to grade COPD severity.

Based on previous pilot data indicating the medians of the questionnaire scores for a comparable group of physicians were 5 and 9 correct answers pre- and post-education, respectively, and assuming a standard deviation of 0.5, to obtain 80% power the sample size of physicians tested must be at least 15. Assuming the average rate of correct diagnosis without spirometry is 20–50% and with spirometry is 90%, and assuming a standard deviation of 50%, to obtain 80% power the sample size of physicians must be at least 15, and the sample size of patients at least 246.

## Results

### Demographics of physicians

A total of 225 eligible internal physicians were recruited from eight secondary hospitals. Questionnaires were administered to 225 physicians, of which 220 were completed, with a recovery rate of 97.8%. 207 questionnaires were qualified and paired according to the study design; 13 were excluded because of unanswered questions.

Demographics of the 207 physicians receiving the one-hour COPD training course are summarized in table 1.

**Table 1:** Demographics of physicians receiving one-hour training course of COPD.

Hospital Name	Physicians(n)	Ages yrs	male gender		Educational degrees* (n)			Medical rank* (n)		
			n	%	L	M	H	L	M	H
Hospital of Rong-cheng	25	39±4	12	48.0	7	18	0	9	13	3
People’s hospital of Hua-yuan county	19	34±7	12	63.2	6	13	0	10	7	2
People’s hospital of An-ren county	26	35±7	17	65.4	7	19	0	8	12	6
People’s hospital of Lin-li county	30	40±4	18	60.0	7	22	1	11	12	7
People’s hospital of Heng-yang county	33	34±8	19	57.6	7	24	2	11	14	8
People’s hospital of Zhu-zhou city hospital	26	43±5	13	50.0	1	24	1	8	15	3
People’s No 2 hospital of Zhu-zhou city	24	33±7	8	33.3	1	21	2	12	8	4
People’s traditional medicine hospital of Ning-xiang county	24	43±3	8	33.3	4	16	4	11	9	4
Total	207	35±7	107	51.7	40	157	10	80	90	37

* L: low level, M: median level, H: high level.

### Physicians’ familiarity with Chinese COPD guideline pre- and post-education

The mean scores of the questionnaire for 207 physicians were 53.1 ± 21.7 and 93.3 ± 9.8 pre- and post-education(*P*<0.05). The physicians in secondary hospitals had limited knowledge about Chinese COPD guideline before the educational intervention, but improved after training. In particular, less than half of physicians correctly answered questions relating to pathogenesis of COPD, severity criteria grading, oxygen therapy for COPD, and indications for non-invasive ventilation. While the average scores of physicians appeared to vary by hospital on the pre-training questionnaire (range 31.6% to 64.8%), the average scores of physicians by hospital on the post-education questionnaire were all greater than 87%.

### Characteristics of the physicians at the ambulatory clinics

18 physicians at 4 secondary hospitals participated in the spirometric intervention at the ambulatory clinics. All had recently attended the educational intervention above. 14 /18 (77.8%) were male; the average age was 42 ± 4 years. All physicians had at least a Bachelor degree (median educational level); 10/18 (55.6%) had median medical rank, and 8/18 (44.4%) had high medical rank.

350 patients were recruited who met the inclusion/exclusion criteria. 307 completed pre- and post-bronchodilator spirometry. Of these patients, 280/307 (67.8%) were male; the average age was 61 ± 7 years; the average cigarette smoking exposure was 37 ± 15 pack-yrs.

### Effect of spirometry on diagnosis of COPD

Spirometry was used as the diagnostic standard. Of the 307 subjects who underwent spirometry, 188/307 had normal spirometry, and 119/307 met criteria for airway obstruction, with a prevalence of COPD of 38.8%. Based on GOLD and the Chinese COPD guideline, 22/119 (7.2%) had mild COPD, 66/119 (38.8%) had moderate COPD, and 31/119 (10.1%) had severe COPD. With regard to chronic respiratory symptoms in the 119 patients with COPD, 75 (63.0%) had chronic cough, 52 (43.7%) had daily sputum production; 20 (16.8%) had wheeze or dyspnea with exertion, and 39 (32.8%) had no chronic symptoms.

Physician assessments of COPD before and after being given spirometry results are shown in Table 2. Without spirometric information, physicians incorrectly diagnosed 34/119 (28.6%) patients with abnormal spirometry as not having COPD, and 38/188 (20.2%) patients with normal spirometry as having COPD. The overall rate of correct diagnoses without spirometry was 76.6% (235/307).

**Table 2:** Physician diagnosis of COPD without and with spirometry.

		COPD Diagnosis by physician without spirometry		COPD Diagnosis by physician with spirometry	
		No	Yes	No	Yes
COPD diagnosis as determined by spirometry	No	150	38	182	6
	Yes	34	85	2	117

Note: Percentage of correct diagnosis without spitometry is 235/307 or 76.6%. Cohen’s kappa coefficient = 0.51 (Almost perfect agreement). Percentage of correct diagnosis with spirometry is 299/307 or 97.4%.Cohen’s kappa coefficient = 0.95 (Almost perfect agreement).

After being given spirometry results, only 2 (1.7%) patients with COPD were misdiagnosed as not having COPD, and 6 (3.2%) patients without COPD were misdiagnosed as having COPD. The overall rate of correct diagnoses with spirometry was 97.39% (299/307). The availability of spirometric information resulted in 69/307 (22.5%) patients having COPD diagnosis correctly changed (*P* value < 0.05).

### Effect of spirometry on evaluating severity of COPD

Tables 3 and 4 demonstrate physician COPD severity grading pre- and post-spirometry testing in the 307 patients. Without spirometry information, physicians correctly graded COPD severity in 58.6% of subjects they identified as having COPD (Cohen’s kappa coefficient = 0.29). With spirometry information, this increased to 93.2% (Cohen’s kappa coefficient = 0.88).

**Table 3:** The coherence between COPD severity determined by spirometry and physician without spirometry.

		COPD severity determined by physician without spirometry results			
		No COPD	Mild	Moderate	Severe
COPD severity by spirometry	No COPD	150	33	4	1
	Mild grade	13	7	2	0
	Moderate grade	20	27	14	5
	Severe grade	0	1	21	9

Notes: Percentage of correct severity grading 180/307 = 58.6%. The under-diagnosis percentage is 82/307 or 20.2%. Cohen’s kappa coefficient = 0.29.

**Table 4:** The coherence between COPD severity determined by spirometry and physician with spirometry.

		COPD severity determined by physician with spirometry results			
		No COPD	Mild	Moderate	Severe
COPD severity by spirometry	No COPD	182	3	2	1
	Mild	2	17	3	0
	Moderate	0	7	57	2
	Severe	0	0	1	30

Notes: Percentage of correct severity grading 286/307 or 93.2%. The under-diagnosis percentage is 10/307 or 3.3%. Cohen’s kappa coefficient = 0.88.

### Effect of spirometry on prescription of COPD

Treatments prescribed to patients before physicians were given spirometry results were compared to treatments prescribed after the results were made available, in the 119 patients who were determined by spirometry to have COPD. Based on the recommendations of GOLD and the Chinese COPD guideline, the percentage of correct COPD treatment prescribed improved from 18.5% (22/119) to 84.0% (100/119) (*P*<0.05). (Table 5). Changes in prescriptive patterns were most evident in the groups of patients with moderate and severe COPD.

**Table 5:** Number of correct COPD prescriptions by physicians with or without spirometry.

COPD diagnosis determined by spirometry		Correct prescription by physicians(n)		
Severity	n	Pre-spirometry(n)	Post-spirometry(n)	*P* value
Mild	22	11	20	*P*<0.05
Moderate	66	8	62	*P*<0.05
Severe	31	3	18	*P*<0.05
In total	119	22	100	

## Discussion

Previous studies[13],[17–19] have focused on the reasons that primary care physicians are unfamiliar with or comply poorly with the Chinese COPD guideline, or on the effect of spirometry on the accuracy of diagnosis and treatment of COPD. This study explored the effects of a short training session followed by access to spirometry on the ability of internal medicine physicians to diagnose and treat COPD in secondary hospitals in China. The study demonstrates that internal medicine physicians in secondary hospitals had gaps in knowledge relating to COPD. Reasons for this may include that the educational background of physicians working in secondary hospitals is limited, ranging from three to five years of medical college after secondary school, and that these physicians in general lack formal medical residency training. This knowledge gap highlights the need for further education initiatives targeted to physicians in primary care settings, particularly in secondary and lower level hospitals. This points out the economic and educational challenges of lengthy training for physicians in a country of huge population and limited resources. We feel that the improvement in the mean scores of the questionnaires performed before and after the short educational intervention in this study demonstrates that this type of limited educational intervention can be effective to improving the basic knowledge of internists.. Such interventions may be more practical and convenient for physicians to master the knowledge required in primary care settings.

While the educational intervention did improve the knowledge base of these primary care physicians relating to COPD and the Chinese COPD guideline, application of such knowledge is limited in the absence of tools like spirometry that can enable diagnosis. Although the physicians performed well on the post-education questionnaires, without spirometry, their ability to diagnose COPD was still limited. Further, the ability to accurately grade COPD severity and to prescribe appropriate treatment was also limited. Education alone is not sufficient; objective data are also necessary to ensure diagnosis and proper treatment.

Limitations to our study include that the sample size was small, thoughresults are comparable to those of previous studies[1]. While there was improvement in knowledge after the educational intervention, physician understanding and performance on the questionnaires post-education as still imperfect, suggesting that the educational intervention could be improved. While spirometry improved the diagnostic accuracy of physicians identifying and grading COPD, there was still some residual misdiagnosis and undertreatment for COPD patients. Whether such interventions could be implemented and would be effective on a large scale remains unanswered.

China is the world’s largest cigarette consumer and producer[1], and tobacco-related diseases, including COPD, are growing health problems in China. For cultural and economic reasons, most Chinese people do not visit a physician unless they are acutely or seriously ill, which leads to underdiagnosis and undertreatment for early or asymptomatic disease. This emphasizes the importance of making such diagnoses when the opportunity arises in the primary care setting. This requires attention to providing both continuing educational opportunities and appropriate tools to primary care physicians. Preventive care and related medical resources are still demanded urgently in most primary care hospitals in China. The Chinese government faces a monumental task in trying to provide adequate medical services to meet the basic needs of its vast population; this extends from physician manpower to all hospitals having access to basic diagnostic equipment.

In conclusion, our study supports short term education in improving physicians’ knowledge relating to COPD in secondary hospitals in China. Providing access to spirometry increased the ability of physicians to diagnose COPD, as well as their accuracy in grading COPD severity.
